# Work–Life Balance: A Modern Concern?

**DOI:** 10.1055/a-2575-1290

**Published:** 2025-05-15

**Authors:** Joon Pio Hong, Jaeyoung Hur

**Affiliations:** 1 Department of Plastic and Reconstructive Surgery, Asan Medical Center, University of Ulsan College of Medicine, Seoul, Republic of Korea; 2 Youngyoung Plastic Surgery Clinic, Seoul, Republic of Korea

When I travel, one of the most common questions I receive is: *How do you balance work and life?* This makes me wonder—why was this not a concern in the past? Has the new generation become spoiled? Do they not understand the true meaning of success, or is there something beyond success itself? To me, hard work is the path to professional success. So why are young people today so preoccupied with this seemingly mundane question?

According to the Cambridge Dictionary, “work–life balance” refers to the amount of time you spend doing your job compared to the time you spend with family and engaging in activities you enjoy. However, achieving balance is not about splitting time evenly between work and leisure; rather, it is about feeling fulfilled and content in both areas. A healthy work–life balance might mean meeting deadlines at work while still making time for friends and hobbies, as well as maintaining proper sleep and a healthy diet.

When I was in training, work felt more like survival than just a job. We rarely had choices—our only option was to strive to be better than the previous generation as a collective. In a way, work was not just a part of life; it *was* life. But times have changed. Life is no longer about mere survival; it is now about personal growth and fulfillment. As societies develop and move beyond poverty and collectivism, the meaning of success has shifted from a shared struggle to an individual pursuit. The focus has transitioned from working out of necessity to working as a means of self-actualization.

However, if your goal is not just to meet deadlines but to become an extraordinary professional, then simply fulfilling basic work expectations is not enough. One must adopt the mindset of past generations—one of sheer hard work and perseverance. In this case, you consciously choose to prioritize work over leisure. But life is not just about leisure; it is a broader concept encompassing the vision and purpose that define one's existence. In a way, the term “work–life balance” can be misleading—a more accurate phrase might be “work–leisure balance.”

The concept of work–life balance varies across countries and cultures. Even within the same country, perspectives differ based on factors such as age, profession, and personal priorities. In fact, even within the same workplace, work–life balance is subjective. Some people choose to invest more time in their careers to achieve professional growth, while others prioritize different aspects of life that require less work-related intensity.

But everything in life comes with tradeoffs. The vision you have for yourself in 10 or 20 years depends on the discipline and perseverance you commit to today. The key is to define your own balance rather than comparing yourself to others. Ultimately, work–life balance is about achieving happiness. Happiness is a personal journey, and whether it is found through professional accomplishments or personal fulfillment outside of work, it requires dedication and effort.

Confucius once said, “Find a job you love, and you will never have to work a day in your life.” So, be conscious and courageous in walking the path that works for you, and do not let others dictate your journey. When you look back 10 or 20 years from now and reflect on your work–life balance, make sure it was a path you deliberately chose—one that aligns with your values, aspirations, and definition of success.

